# Intranasally Administered Antigen 85B Gene Vaccine in Non-Replicating Human *Parainfluenza* Type 2 Virus Vector Ameliorates Mouse Atopic Dermatitis

**DOI:** 10.1371/journal.pone.0066614

**Published:** 2013-07-03

**Authors:** Hiroshi Kitagawa, Mitsuo Kawano, Keiichi Yamanaka, Masato Kakeda, Kenshiro Tsuda, Hiroyasu Inada, Misao Yoneda, Tadashi Sakaguchi, Akina Nigi, Koumei Nishimura, Hiroshi Komada, Masato Tsurudome, Yasuhiro Yasutomi, Tetsuya Nosaka, Hitoshi Mizutani

**Affiliations:** 1 Department of Dermatology, Mie University, Graduate School of Medicine, Tsu, Mie, Japan; 2 Department of Microbiology and Molecular Genetics, Mie University, Graduate School of Medicine, Tsu, Mie, Japan; 3 Department of Pathology, Faculty of Pharmaceutical Science, Suzuka University of Medical Science, Suzuka, Mie, Japan; 4 Department of Clinical Nutrition, Graduate School of Suzuka University of Medical Science, Suzuka; Mie, Japan; 5 Laboratory of Immunoregulation and Vaccine Research, Tsukuba Primate Research Center, National Institute of Biomedical Innovation, Tsukuba, Ibaraki, Japan; University Heart Center Freiburg, Germany

## Abstract

Atopic dermatitis (AD) is a refractory and recurrent inflammatory skin disease. Various factors including heredity, environmental agent, innate and acquired immunity, and skin barrier function participate in the pathogenesis of AD. T -helper (Th) 2-dominant immunological milieu has been suggested in the acute phase of AD. Antigen 85B (Ag85B) is a 30-kDa secretory protein well conserved in *Mycobacterium* species. Ag85B has strong Th1-type cytokine inducing activity, and is expected to ameliorate Th2 condition in allergic disease. To perform Ag85B function in vivo, effective and less invasive vaccination method is required. Recently, we have established a novel functional virus vector; recombinant human *parainfluenza* type 2 virus vector (rhPIV2): highly expressive, replication-deficient, and very low-pathogenic vector. In this study, we investigated the efficacy of rhPIV2 engineered to express Ag85B (rhPIV2/Ag85B) in a mouse AD model induced by repeated oxazolone (OX) challenge. Ear swelling, dermal cell infiltrations and serum IgE level were significantly suppressed in the rhPIV2/Ag85B treated mouse group accompanied with elevated IFN-γ and IL-10 mRNA expressions, and suppressed IL-4, TNF-α and MIP-2 mRNA expressions. The treated mice showed no clinical symptom of croup or systemic adverse reactions. The respiratory tract epithelium captured rhPIV2 effectively without remarkable cytotoxic effects. These results suggested that rhPIV2/Ag85B might be a potent therapeutic tool to control allergic disorders.

## Introduction

Atopic dermatitis (AD) is a refractory and recurrent inflammatory skin disease. Heredity, environmental agent, immunity, and skin barrier function participate in the pathogenesis of AD. AD symptoms are triggered by various non-specific or specific allergic reactions. The cytokine pattern of AD, especially in the acute phase skin lesion is Th2-type cytokine dominant [1]. The barrier disrupted skin in AD is easily permitted the percutaneous entry of environmental allergens that strongly promotes Th2 immunological responses [2]. Th2 cells as well as T regulatory cell (Treg) subsets play key roles in development of AD. Patients with AD have significantly increased numbers of peripheral blood Treg compared with healthy controls, which is correlated with disease activity in AD [3], [4]. This suggests involvement of some self regulation system in immune responses in AD [5].

Repeated elicitation with hapten such as oxazolone (OX) on the ear of BALB/c mice develops immediate type responses with late phase reactions followed by delayed type hypersensitivity responses. This accompanied with balance shift of cytokines in the lesional skin from Th1 to Th2 type [6], and has been utilized as mouse AD.

Ag85B is 30-kDa major secretory protein well conserved in *Mycobacterium* species [7]. The studies for the tuberculosis vaccine revealed strong activities of Ag85B in priming naïve T cells for Th1 effector cells under the appropriate conditions, and induction of strong Th1-type immune responses in mice as well as in humans [8], [9]. Recently we reported that plasmid DNA vaccination encoding Ag85B derived from M. *kansasii* inhibits immediate-type hypersensitivity responses with Treg induction in skin [10], and a combined vaccination with heat-killed BCG followed by Ag85B also suppressed skin eczematous reactions in AD model mice by inducing Treg [11].

Human parainfluenza type 2 virus (hPIV2) is one of the human respiratory pathogens and a member of the genus Rubulavirus of the family Paramyxoviridae in the order Mononegavirales, possessing a non-segmented and negative-stranded RNA genome of 15,654 nucleotides. The genome of hPIV2 encodes 7 mRNAs [12]–[14] and has about 60-nt leader sequence at 3′ end and about 20-nt noncoding trailer sequence. The gene order is 3′ (leader)-NP-V/P-M-F-HN-L-(trailer)-5′. The coding proteins are the nucleocapsid (NP), the V (V) and phospho (P), the matrix (M), the fusion (F), the haemagglutinin-neuraminidase (HN), and the polymerase protein (L). The genomic RNA of the virus: viral RNA (vRNA) is encapsidated with the NP proteins, and the nucleocapsids are associated with the P and L proteins to form the ribonucleoprotein complex. In paramyxovirus particles, vRNA is enclosed by the viral envelope composed of a cellular lipid bilayer and two envelope glycoproteins, HN and F, which are integral transmembrane proteins mediating virus attachment and cell fusion, respectively [15]. M protein underlies the lipid bilayer to ensure the structural integrity of the viral particles and is essential for interactions between the viral envelope and the RNP complex [15]. This association leads to the budding and release of viral particles from the cell surface [15].

Recently, as technology advances in reverse genetics [16], hPIVs offer several advantages as a vaccine vector. hPIVs efficiently infect the respiratory tract but don’t spread far beyond it, which is an important safety factor. hPIV-based vectors have proven the effect in inducing local and systemic immunity against a number of foreign antigens [17]. hPIVs infect to various cell types and cause little cytopathic effects. Moreover, they replicate exclusively in the cytoplasm of infected cells, don’t have a DNA phase during their life cycle and can thus avoid the possibility of integration of foreign genes into the host DNA genome [18].

In the present study, we utilized newly engineered rhPIV2: replication-deficient rhPIV2 vector. rhPIV2 lacks M gene that is an essential gene for virus particle formation by insertion of two stop codons. This alteration might support much safer application to animals than original proliferating virus vector. We first investigated efficiency of rhPIV2 vaccine vector expressing enhanced green fluorescence protein (EGFP) gene (rhPIV2/EGFP) in infection and expression in vitro and in vivo. Then, we evaluated effectiveness of the vaccination pathways: subcutaneous or intranasal administration of rhPIV2 expressing Ag85B gene (rhPIV2/Ag85B) in a mice AD model induced by repeated hapten challenge.

## Materials and Methods

### Animals

BALB/c 6-week old male mice were purchased from Japan SLC Co. (Shizuoka, Japan) and used at 7-week. Animal care was performed according to ethical guidelines, and approved by the Institutional Board Committee for Animal Care and Use of Mie University.

### Construction of rhPIV2/Ag85B and rhPIV2/EGFP

**Figure 1:**
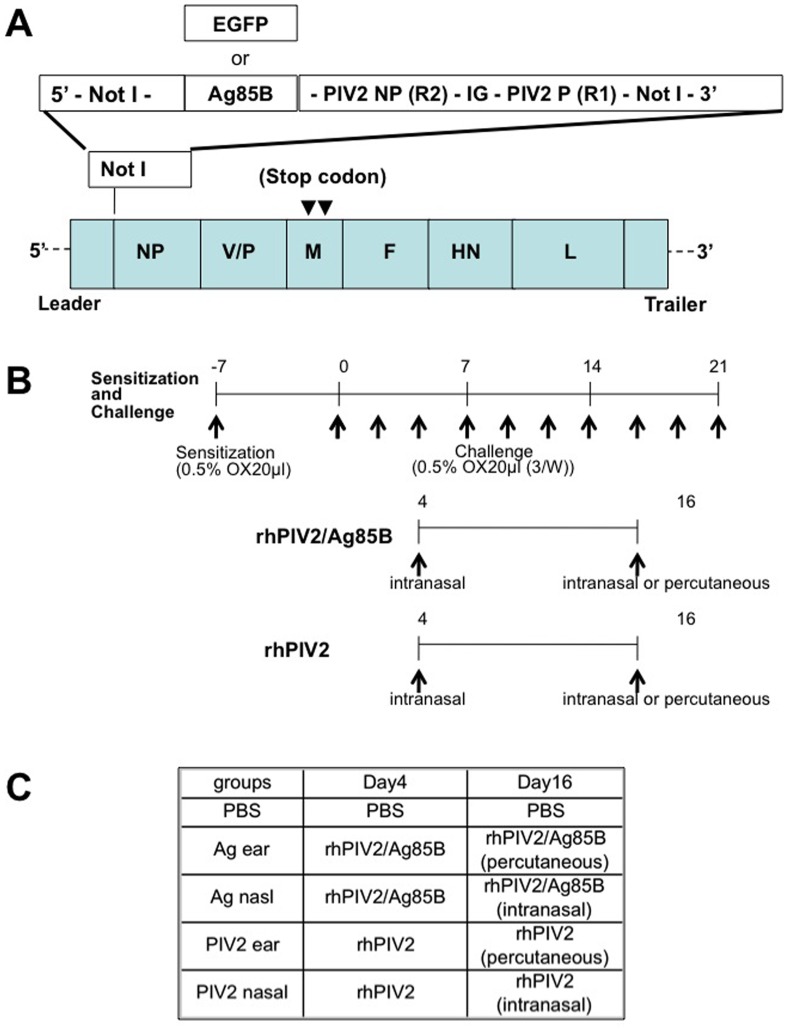
Schematic diagram of constructs and strategy used in this study. **A.** The constructs of recombinant hPIV2/EGFP and hPIV2/Ag85B. The EGFP or Ag85B gene open reading frame was engineered to be flanked by hPIV2-specific gene end of NP gene (R2), intergenic sequence (IG), and gene start (R1) transcriptional signal of V/P gene. It was inserted into a cloned cDNA of the hPIV2 antigenome at a Not I site that had been engineered to be at 5′-noncoding region of NP gene. A genomic nucleotide length divisible by six (the rule of six) was maintained. For generating of replication-deficient virus, two stop codons (▾) were introduced on the M gene. **B.** Schedule for the development of a hapten-induced atopic dermatitis model and vaccination of rhPIV2/Ag85B. Mice were initially sensitized with 20 µl of 0.5% OX solution to their right ear 7 days prior to the first challenge (day -7) and then 20 µl of 0.5% OX solution was repeatedly applied on the right ear 3 times per week from day 0 until day 21. Mice were inoculated intranasally with 20 µl (5×10^6^ TCID_50_) of rhPIV2/Ag85B or rhPIV2 on day 4. rhPIV2 vector or phosphate buffered saline (PBS) were also applied as controls. On day 16, mice were vaccinated again intranasally or subcutaneously with PBS, rhPIV2 or rhPIV2/Ag85B. **C.** Summarized schedule of the experimental groups.

rhPIV2/Ag85B and rhPIV2/EGFP was constructed according to the method reported previously, except for methods of the supply of T7 and hPIV2 RNA polymerases (NP, P, L). In brief, to generate replication-deficient rhPIV2 vector, two nucleotides change [ATG to TAG (position of 89aa) and AAG to TAG (259aa)] were introduced into the M frame of the plasmid pPIV2, a full-length cDNA copy of hPIV2 anti-genome [19] (Fig. 1A). Consequently, the 6 n length cDNA of Ag85B or EGFP, followed by transcriptional end sequence of NP gene (R2), intergenic sequence (IG), and transcriptional start signal of V/P gene (R1) ([20] was synthesized by PCR using appropriate primers), was inserted into a Not I site of the plasmid DNA encoding the replication-deficient rhPIV2 genome described above. Then, the viruses (rhPIV2/Ag85B and rhPIV2/EGFP) were recovered by co-transfection of each anti-genomic plasmid and plasmids expressing the NP, P, M and L, each cloned in a mammalian gene expression vector (pCAGGS) [21] into BSR7/5 cells expressing T7 RNA polymerase [22]. The cells were harvested, and then co-cultured with fresh Vero cells every 48 hr. Approximately 90% of the cells showed syncytia formation in the 10^th^ co-cultured cells, and its state was maintained in further co-culture. Furthermore, for virus propagation, Cos7 cells were transfected with the plasmid expressing M and co-cultured with above-mentioned 10^th^ cells. The supernatant was centrifuged at 9,000 g for 12 h at 4°C. The virus pellet was suspended in Opti-MEM (Invitrogen, Carisbad, CA, USA). The virus titers were determined by CPE method using Vero cells, and were expressed as 50% tissue culture infectious dose (TCID_50_).

### In vitro and in vivo Infection of rhPIV2 Vector Expressing EGFP

HaCat cells (Cell Line Service, Eppelheim, Germany) were cultured in Dulbecco’s MEM supplemented with 5% (v/v) FBS, 2.0 mM L-glutamine, 100 U/ml penicillin, and 100 mg/ml streptomycin. HaCat cells were seeded one day before the transduction at 1×10^6^ cells/ml (1 ml/well) in 6-well culture plates (Costar, NY, USA). The cells were incubated for 8 hours at 37°C in a 5% CO_2_ atmosphere. The next day, the media was removed and 1 ml of the rhPIV2/EGFP viruses were added to the cells to be adjusted to 1×10^6^ TCID_50_. Two hours after infection, the media was removed and fresh culture media was supplemented to the cells. After 3 days culture, each well was observed by fluorescence microscopy.

At the next step, 20 µl of concentrated rhPIV2/EGFP (5×10^6^ TCID_50_) were administered into the cavity of the nose of the mice. Four days after infection, the respiratory tract and lung were sampled, embedded in Tissue-Tek OCT compound (Miles, Elkhart, USA), frozen in liquid nitrogen, and cut into 7 µm-thick sections. Sections were examined and recorded by fluorescence microscopy.

### Sensitization and Challenge Schedule

Repeated hapten sensitization and challenge system was introduced in this experiment. OX (Sigma, St. Louis, MO) was dissolved in acetone/olive oil (1∶1). As shown in Fig. 1B, mice were initially sensitized by pasting 20 µl of 0.5% OX solution to their right ear 7 days prior to the first challenge (day -7) and then 20 µl of 0.5% OX solution was repeatedly applied on the right ear 3 times per week from day 0 until day 21. Repeated application of OX causes delayed type hypersensitivity followed by immediate-type and late phase reaction. For vaccination, mice were infected intranasally under general anesthesia with 5×10^6^ TCID_50_ of the virus in a 20 µl inoculum or phosphate-buffered saline (PBS) on day 4. On day 16, mice were vaccinated again with PBS, rhPIV2/Ag85B or control rhPIV2 vector intranasally or subcutaneously to the pinna skin (Fig. 1B,C). Ear swelling was measured with thickness gauge calipers before and 6 hours after last OX challenge on day 21. Blood and pinna skins were also sampled.

### Histopathological Study

The ear skins were sampled at six hours after last OX challenge on day 21. Samples were fixed in 10% neutral buffered formaldehyde and embedded in paraffin. Histological sections were of 6 mm thickness and stained with hematoxylin & eosin (H&E).

### Analysis of Cytokine mRNA Expression in Mouse Ear

The mRNA was extracted from the mouse ear using Isogen (Nippon Gene, Tokyo, Japan) according to the manufacturer’s instructions: One ml of homogenate was mixed with 200 µl of chloroform, and then centrifuged. The aqueous phase was separated and mixed with 0.5 ml of 2-propanol (Nacalai Tesque, Kyoto, Japan) to precipitate RNA. After centrifuging, the precipitate was washed with 70% ethanol (Nacalai Tesque) and the RNA was suspended in 40 µl of RNase-free water. The concentration of RNA was measured at 260 nm absorbent, and the quality was confirmed by electrophoresis. cDNA was synthesized from 2 µg of mRNA using an archive kit (Applied Biosystems, Foster City, CA, USA) according to the manufacturer’s protocol. Real time quantitative reverse transcription-polymerase chain reaction (RT-PCR) was performed to measure transcriptional activity in skin lesions. A 25 µl reaction mixture containing 1 µg of cDNA, 900 nM of each primer, and 250 nM of TaqMan probe was mixed with 12.5 µl of TaqMan Master Mix (AB). Quantitative RT-PCR for cytokine transcripts was performed using prequalified primers and probes for IL-2, IL-4, IL-10, IL-17A, MIP2, TNF-α, TGF-β, IFN-γ and GAPDH (AB). The ΔCt method was used to standardize the transcripts to GAPDH, and the ratio to that of control mice was calculated.

### Immunohistochemistry

The ear skins sampled on day 21 were snap-frozen, and the frozen sections prepared at 7 µm thickness were subjected to a blocking procedure with 5% normal goat serum (Vector Laboratories, Burlingame, CA). Sections were then incubated with FITC-conjugated rat anti-mouse CD4 antibody (Beckman Coulter) and PE conjugated anti-mouse FoxP3 antibody (BioLegend), and examined under Fluoview FV1000 laser scanning confocal microscopy (Olympus, Tokyo, Japan). Skin infiltrating CD4^+^ T cells and FoxP3^+^CD4^+^ T cells were counted at x100 field, and the numbers in 10 randomly chosen fields of five samples were evaluated.

### Measurement of Serum IgE

Serum IgE level was determined by a sandwich enzyme-linked immunosorbent assay (Yamasa, Tokyo, Japan) according to the manufacturer’s instructions. Optical density of each well was determined by using a microplate reader (Multiscan JX, Thermo Electron, Yokohama, Japan).

### Statistical Analysis

Statistical analysis was performed using Mann-Whitney U-test. P<0.05 was considered as significant.

## Results

### rhPIV2/EGFP Infection in vitro and in vivo

**Figure 2:**
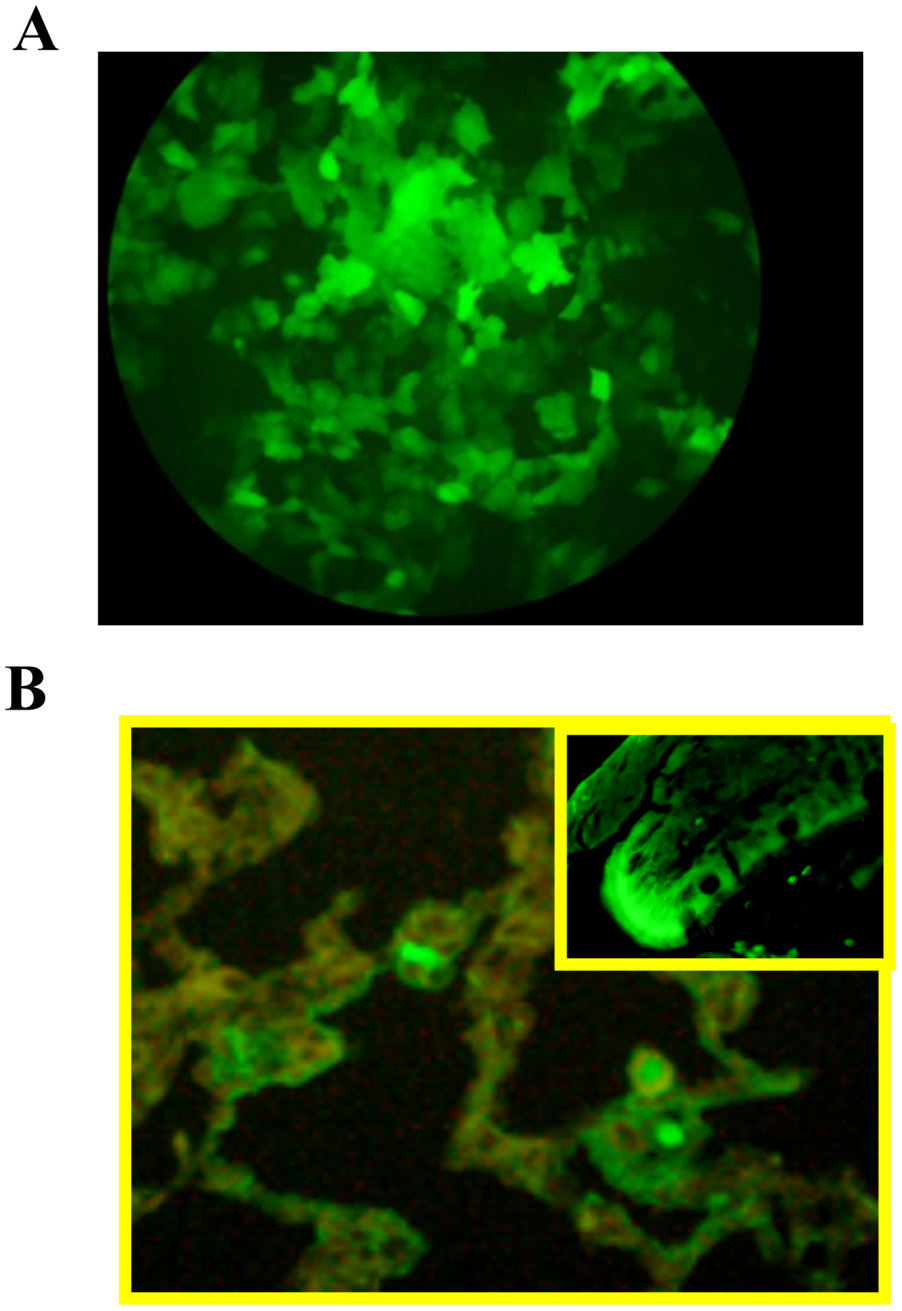
Expression of EGFP from rhPIV2/EGFP. **A.** HaCat cells were infected with rhPIV2/EGFP at an MOI of 0.5. Three days after, EGFP was clearly visualized using a fluorescence microscopy (x100). **B.** The rhPIV2/EGFP (5×10^6^ TCID_50_) were administered to a wild type BALB/c mice intranasally EGFP was visualized clearly in the airway epithelial cells 4 days after administration (x200, upper right box, x400).

To investigate expression levels of the inserted gene in rhPIV2 in vitro, HaCat cells were infected with rhPIV2/EGFP at an MOI of 0.5 and were examined directly using a fluorescence microscopy. The EGFP from rhPIV2/EGFP was highly expressed in HaCat cells, and remarkable fluorescence extended to nearly all the cells in spite of low MOI (Fig. 2A). Then, to evaluate the gene expression in vivo, mice were intra-nasally inoculated with rhPIV2/EGFP (5×10^6^ TCID_50_), and the intense EGFP expression was revealed in the lung epithelium of the mice (Fig. 2B).

### Cutaneous Manifestations

**Figure 3:**
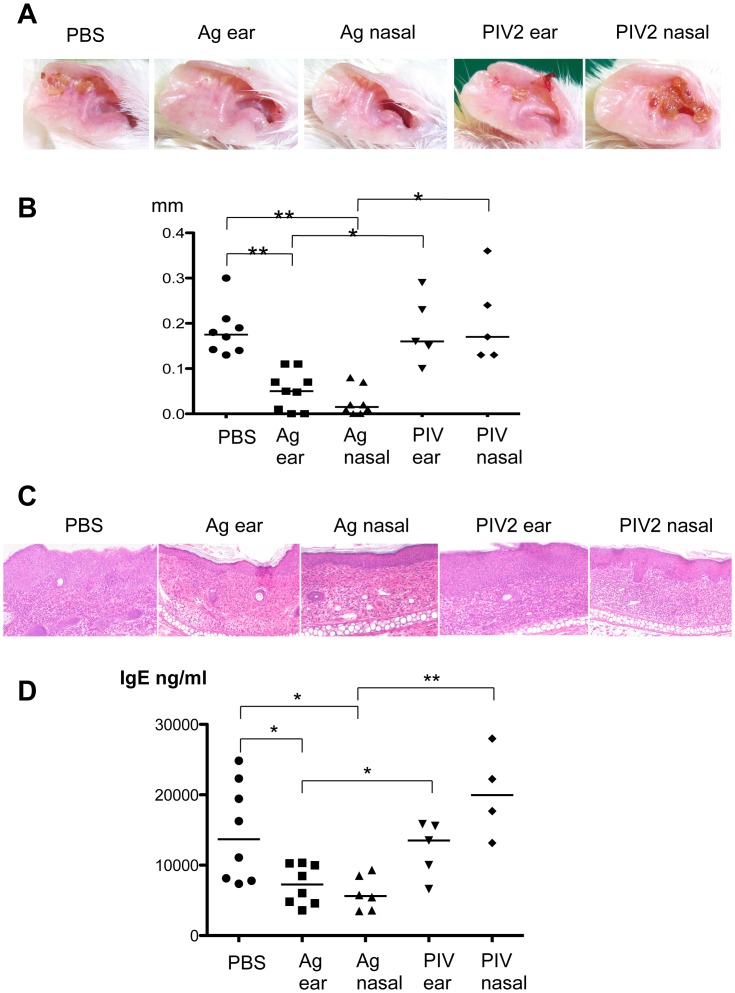
Anti-inflammatory effects of vaccination with rhPIV2/Ag85B. **A.** Clinical manifestation of the ear skin at 6 hours after OX challenge on day 21. The control groups (PBS, PIV2 ear, and PIV2 nasal on the panel) showed severe edema with erythema, however the intranasal and/or subcutaneous administration of the rhPIV2/Ag85B (Ag nasal and Ag ear on the panel, respectively) clearly reduced skin reactions in OX-sensitized mice. **B.** Ear thickness measured before and 6 hours after each OX application on day 21. The ear swelling was suppressed significantly in rhPIV2/Ag85B treated groups in two ways compared to those in the placebo treated groups. (*P<0.05, **P<0.01.) **C.** Histopathological changes of the ear skin obtained on day 21 in paraffin embedded sections stained with hematoxylin and eosin. The placebo treated groups (PBS, PIV2 ear and PIV2 nasal on the panel) revealed marked inflammatory reactions with acanthosis and ulceration in epidermis, and marked edema with cellular infiltration including mononuclear cells and neutrophils in the dermis. The skin infiltration of inflammatory cells and epidermal thickness were decreased in rhPIV2/Ag85B treated group (Ag85B ear and Ag85B nasal on the panel). Original magnification × 100. **D.** Plasma IgE levels on day 21. Plasma IgE level was decreased in rhPIV2/Ag85B treated groups (Ag ear and Ag nasal). *P<0.05, **P<0.01.

To evaluate the clinically relevant therapies, mice were treated following the strategy shown in Fig. 1B. Ear lobes of the rhPIV2 (vector alone) or PBS-treated mice developed severe edematous erythema with exudation and erosion at 6 hours after OX challenge on day 21. However, rhPIV2/Ag85B-treatment reduced dermatitis in both of the intra-nasal and subcutaneous application groups (Fig. 3A). Ear swelling was dramatically suppressed in both of the rhPIV2/Ag85B-treated mice compared to PBS or rhPIV2 treated mice (Fig. 3B).

### Histopathological Findings

PBS or rhPIV2-treated mice showed marked inflammatory reactions with acanthosis and ulceration in epidermis, and marked edema with cellular infiltration including mononuclear cells and neutrophils in the dermis. Both of the intra-nasal and subcutaneous rhPIV2/Ag85B application successfully reduced inflammatory cell infiltration and epidermal thickness (Fig. 3C).

### Serum IgE Levels

High levels of IgE were detected in sera from PBS or rhPIV2-treated mice. On the other hand, the IgE levels in the sera from Ag85B-treated mice by two ways were suppressed significantly (Fig. 3D).

### Cytokines mRNA Expression in the Ear Skins

**Figure 4:**
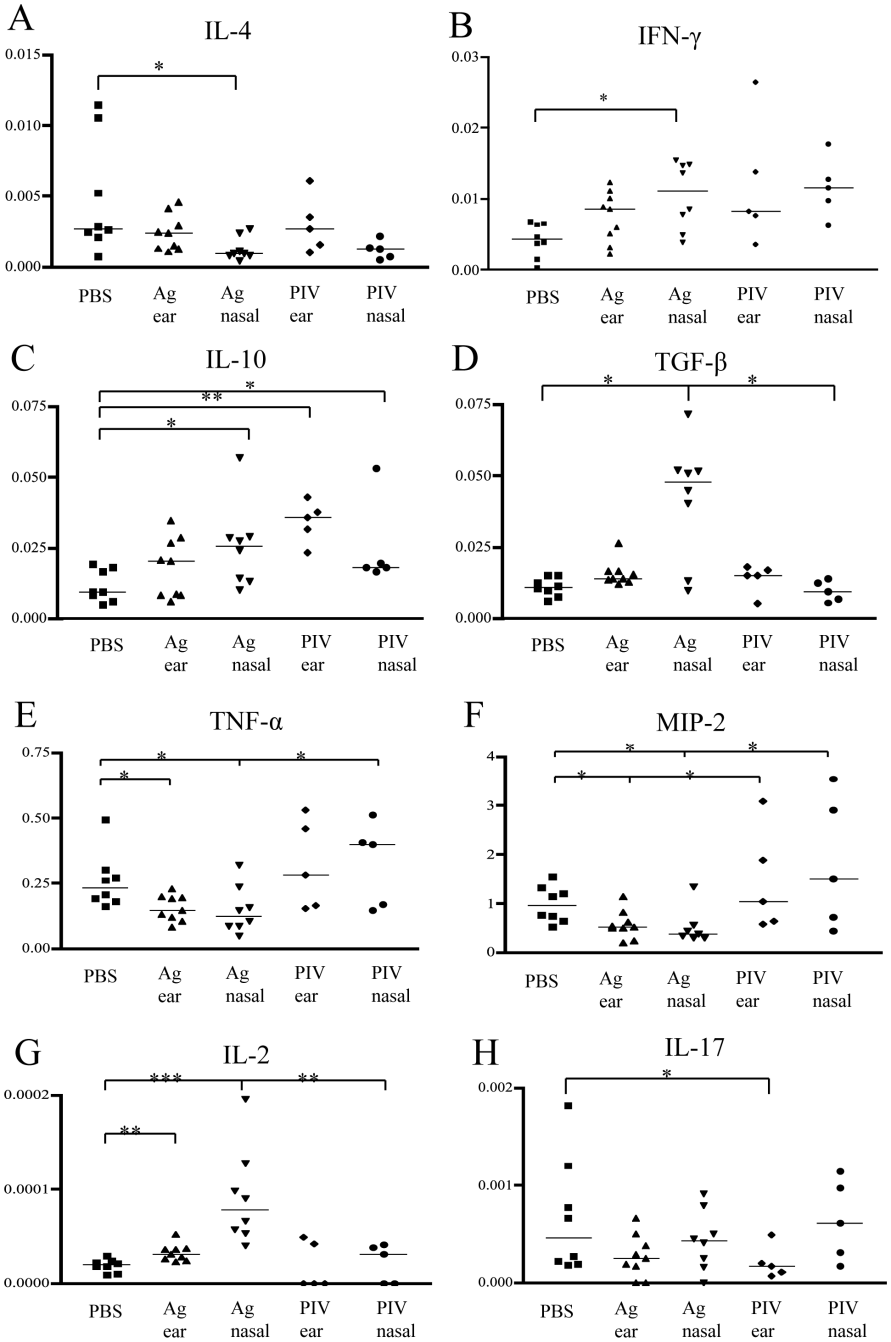
Changes in cytokines mRNA expression levels in the ears of AD mice by vaccination of rhPIV2/Ag85B. Cytokines: IL-4 (panel A), IFN-γ (panel B), IL-10 (panel C), TGF-β (panel D), TNF-α (panel E), MIP2-α (panel F), IL-2 (panel G), IL-17 (panel H), mRNA expression in the ear lesions measured with Quantitative RT-PCR. Expressions of IL-4, TNF-α and MIP2-α mRNA were significantly decreased in the ear skin treated with intra-nasally rhPIV2/Ag85B treated group compared to those of control groups. Meanwhile, the expression levels of mRNA of IFN-γ, IL-10, TGF-β and IL-2 were significantly elevated in rhPIV2/Ag85B intra-nasally treated group compared to those of control groups. *P<0.05, **P<0.01, ***P<0.001.

Expression of IL-4 mRNA was significantly decreased in the ear skin of intra-nasally rhPIV2/Ag85B treatment group compared to that of PBS treated mice (Fig. 4A). In clear contrast, IFN-γ mRNA expression was significantly increased in rhPIV2/Ag85B intra-nasally treated group (Fig. 4B). As expected, IL-10 levels were significantly increased in intra-nasally treated with rhPIV2/Ag85B and rhPIV2-vector groups (Fig. 4C). Surprisingly, TGF-β expression is remarkably elevated in the intra-nasal rhPIV2/Ag85B group (Fig. 4D). mRNA expressions of TNF-α and MIP-2 were significantly decreased in both of intra-nasally and subcutaneously rhPIV2/Ag85B treated groups compared with PBS or vector treated group (Fig. 4E, F). The expression of IL-2 mRNA was also significantly elevated in both of the rhPIV2/Ag85B intra-nasally and subcutaneously treated groups (Fig. 4G). No obvious suppression in IL-17 mRNA expression was detected in rhPIV2/Ag85B group (Fig. 4H).

### Immunostaining for Tregs in the Inflamed Ear Skin Lesions

**Figure 5:**
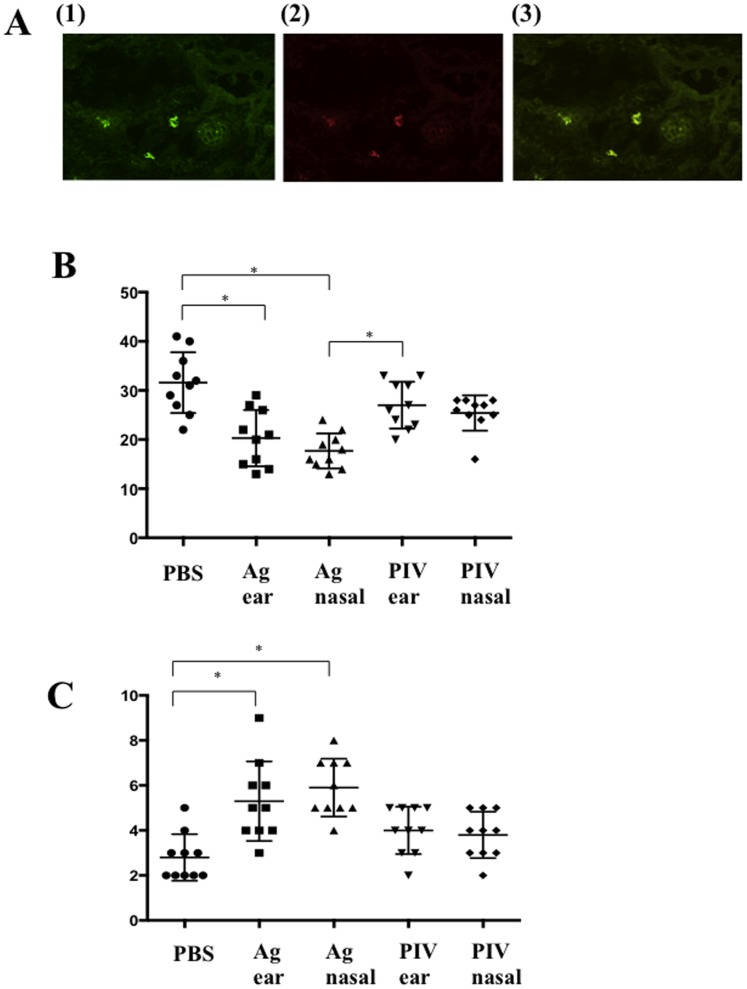
Immunostaining for Tregs in the inflamed ear skin lesions. **A.** CD4^+^ T cells are displayed with green fluorescence (1), and Foxp3^+^ T cells are with red (2). Merged yellow color means Foxp3^+^CD4^+^ T cells (3) (x100). **B.** The number of skin infiltrating CD4^+^ T cells is less in Ag nasal group and Ag ear group compared to that of PBS-treated group. Although it does not reach statistical significance, CD4^+^ T cell number is less in Ag nasal group compared to that of Ag ear group. **C.** The number of Foxp3^+^CD4^+^ T cells in the inflamed ear skin is significantly increased in both of the intra-nasal and ear-subcutaneous rhPIV2/Ag85B application groups.

As shown in Fig. 5A, CD4^+^ T cells are displayed with green fluorescence, and Foxp3^+^ T cells are with red. Merged yellow color means Foxp3^+^CD4^+^ T cells. The skin infiltrating CD4^+^ T cells are significantly decreased in Ag nasal group and Ag ear group compared to that of PBS-treated group. Although it does not reach the significance, the CD4^+^ T cells number is less in Ag nasal group compared to Ag ear group (Fig. 5B). The Foxp3^+^CD4^+^ T cells are significantly increased in both of Ag nasal and Ag ear groups (Fig. 5C).

## Discussion

Immune system is finely controlled on the balance of four main subsets of Th1, Th2, Th17, and Treg [5] cells. AD, especially in its acute phase, is a disease that Th2 cells are dominantly involved in the pathogenesis. In fact PBMCs from patients with AD have increased production of IL-4, IL-5, and IL-13 with limited capacity in production of IFN-γ [23]–[25]. Repeated elicitation of OX on mice ear shifts the cutaneous Th1 cytokine milieu to Th2, which represents the characteristic immunological features of AD. Immunotherapy for AD has some different options to correct the imbalance of the shifted cytokine profile. In the present study, we investigated effects of vaccination using replication-deficient rhPIV2 vector expressing Ag85B gene to mouse AD model. BCG is known as a strong Th1 response modifier; however, it has a risk for granuloma formation. To avoid granuloma formation, non-wax protein antigen is required. Ag85B is a conserved protein in mycobacterial species and can elicit a strong Th1-type immune response [8]. Therefore, Ag85B has been used as immune-modulator to control acute AD lesions or asthma. Ag85B DNA vaccine suppressed airway inflammation in a murine model of asthma [26]. Furthermore, resent studies suggested that Ag85B vaccination promotes Th1-type immune responses as well as Treg responses. Administration of Ag85B showed therapeutic effects to Th2-type cytokine mediated acute phase AD models by inducing regulatory T cells [10].

Selection of the vector and its application pathway has importance in successful DNA vaccination therapy. We selected hPIV2 as a potential vector for Ag85B vaccination. hPIVs are human respiratory pathogens, and the most distinctive clinical feature of infection of hPIVs is croup (i.e., laryngotracheobronchitis or swelling around the vocal chords and other parts of the upper and middle airway). Among hPIVs, hPIV1 and hPIV3 are the major cause of croup in children, whereas hPIV2 is rarely identified as a clinical pathogen. Therefore, hPIV2 has been suspected as less virulent and cytotoxic virus. hPIV2 enters the cell by cell fusion at the plasma membrane, and replicates exclusively in the cytoplasm, and buds at the plasma membrane. Therefore, hPIV2 has no risk for integration in the host genome, not like retrovirus. In addition, since hPIV2 has a non-segmented and negative-stranded RNA genome, there is no antigenic shift among RNA segments, not like influenza viruses. Using technology of advanced reverse genetics [20], we constituted replication-deficient hPIV2 vector with additional advantage as a highly safe virus vector. To confirm the target and effective infection of rhPIV2 vector, we inoculated rhPIV2/EGFP to HaCat cells. HaCat cells successfully expressed EGFP up to 7 days post-infection (pi). Also, BALB/c mice intranasally single-administrated with rhPIV2/EGFP showed intense EGFP expression in the airway epithelial cells. These results strongly support activities of long-term high-level expression of the exogenous gene and efficiency of rhPIV2 in vivo.

In the present study, AD symptoms including ear swelling at late phase reaction were significantly suppressed in rhPIV2/Ag85B treated groups in both of intra-nasal and subcutaneous administration. Inflammatory cell infiltration including mast cells and eosinophils in the lesional skin was also suppressed. In the cytokine profile, mRNA expression of IFN-γ and IL-2 in the ear skins was significantly increased. Interestingly, IL-4 mRNA was significantly reduced in intranasal rhPIV2/Ag85B treated groups. In IL-4 suppression and IFN-γ induction, intra-nasal application showed stronger effects compared with subcutaneous application. hPIV2 is a virus infectious to the respiratory tract mucosa, and therefore more effective capture of rhPIV2/Ag85B by respiratory epithelium compared with that of skin resident cells is reasonable. In addition, the skin derived anti-infectious molecule, horny layer proteases and epithelial skin barrier might decrease efficiency of rhPIV2.

Treg induction in the effects of rhPIV2/Ag85B therapy has importance. Present study unveiled augmentation of TGF-β and IL-10 expression by intranasal rhPIV2/Ag85B. TGF-β and IL-10 have been described as critical regulatory cytokines produced by Treg. In fact in the current experiment, the numbers of skin infiltrating CD4^+^ T cells are decreased in the nasal application and ear skin application groups accompanied with increased FoxP3^+^ Treg population. A heat-killed Mycobacterium vaccae (*M. vaccae*) gives rise to allergen specific regulatory T cells that produce IL-10 and TGF-β, which confer the protection against airway inflammation [27]. Recently TGF-β was proved to suppress GATA-3 function through Sox4 signal, and TGF-β controls Th2 cell-mediated inflammation [28]. In addition, it is crucial that PIV2 itself has some effects in induction of Treg without obvious effects in clinical manifestation and Th1/Th2 balance.

In conclusion, the respiratory tract epithelium captured rhPIV2 effectively without remarkable cytotoxic effects. The treatment with rhPIV2/Ag85B especially by trans-nasal mucosa approach ameliorates OX-induced AD model by altering Th2/Th1 cytokine balance with induction of regulatory cytokines induction. Thus, nasal rhPIV2/Ag85B vaccination is a novel, less invasive and useful therapeutic approach for AD and related allergic disorder.

## References

[1] Grewe M, Bruijnzeel-Koomen CA, Schopf E, Thepen T, Langeveld-Wildschut AG, et al (1998) A role for Th1 and Th2 cells in the immunopathogenesis of atopic dermatitis. Immunol Today 19: 359–361. 9709503 10.1016/s0167-5699(98)01285-7

[2] Kondo H, Ichikawa Y, Imokawa G (1998) Percutaneous sensitization with allergens through barrier-disrupted skin elicits a Th2-dominant cytokine response. Eur J Immunol 28: 769–779. 9541570 10.1002/(SICI)1521-4141(199803)28:03<769::AID-IMMU769>3.0.CO;2-H

[3] Ou LS, Goleva E, Hall C, Leung DY (2004) T regulatory cells in atopic dermatitis and subversion of their activity by superantigens. J Allergy Clin Immunol 113: 756–763. 15100684 10.1016/j.jaci.2004.01.772

[4] Ito Y, Adachi Y, Makino T, Higashiyama H, Fuchizawa T, et al (2009) Expansion of FOXP3-positive CD4+CD25+ T cells associated with disease activity in atopic dermatitis. Ann Allergy Asthma Immunol 103: 160–165. 19739430 10.1016/S1081-1206(10)60170-6

[5] Yamanaka K, Mizutani H (2011) The role of cytokines/chemokines in the pathogenesis of atopic dermatitis. Current problems in dermatology 41: 80–92. 21576949 10.1159/000323299

[6] Kitagaki H, Ono N, Hayakawa K, Kitazawa T, Watanabe K, et al (1997) Repeated elicitation of contact hypersensitivity induces a shift in cutaneous cytokine milieu from a T helper cell type 1 to a T helper cell type 2 profile. J Immunol 159: 2484–2491. 9278342

[7] Nagai S, Wiker HG, Harboe M, Kinomoto M (1991) Isolation and partial characterization of major protein antigens in the culture fluid of Mycobacterium tuberculosis. Infect Immun 59: 372–382. 1898899 10.1128/iai.59.1.372-382.1991 PMC257751

[8] Takatsu K, Kariyone A (2003) The immunogenic peptide for Th1 development. Int Immunopharmacol 3: 783–800. 12781696 10.1016/S1567-5769(02)00209-6

[9] Russo DM, Kozlova N, Lakey DL, Kernodle D (2000) Naive human T cells develop into Th1 effectors after stimulation with Mycobacterium tuberculosis-infected macrophages or recombinant Ag85 proteins. Infect Immun 68: 6826–6832. 11083801 10.1128/iai.68.12.6826-6832.2000 PMC97786

[10] Mori H, Yamanaka K, Matsuo K, Kurokawa I, Yasutomi Y, et al (2009) Administration of Ag85B showed therapeutic effects to Th2-type cytokine-mediated acute phase atopic dermatitis by inducing regulatory T cells. Arch Dermatol Res 301: 151–157. 18633632 10.1007/s00403-008-0873-y

[11] Kakeda M, Yamanaka K, Kitagawa H, Tsuda K, Akeda T, et al. Heat-killed bacillus Calmette-Guerin and Mycobacterium kansasii antigen 85B combined vaccination ameliorates dermatitis in a mouse model of atopic dermatitis by inducing regulatory T cells. Br J Dermatol 166: 953–963. 22136598 10.1111/j.1365-2133.2011.10763.x

[12] Kawano M, Bando H, Ohgimoto S, Okamoto K, Kondo K, et al (1990) Complete nucleotide sequence of the matrix gene of human parainfluenza type 2 virus and expression of the M protein in bacteria. Virology 179: 857–861. 2173264 10.1016/0042-6822(90)90155-k

[13] Kawano M, Bando H, Ohgimoto S, Kondo K, Tsurudome M, et al (1990) Sequence of the fusion protein gene of human parainfluenza type 2 virus and its 3′ intergenic region: lack of small hydrophobic (SH) gene. Virology 178: 289–292. 2167555 10.1016/0042-6822(90)90406-h

[14] Ohgimoto S, Bando H, Kawano M, Okamoto K, Kondo K, et al (1990) Sequence analysis of P gene of human parainfluenza type 2 virus: P and cysteine-rich proteins are translated by two mRNAs that differ by two nontemplated G residues. Virology 177: 116–123. 2162103 10.1016/0042-6822(90)90465-4

[15] Lamb RA, Kolakofsky D (2001) Paramyxoviridae: the viruses and their replicatio. In: Knipe DM, Howley PM, editors. In Fields Virology. Fourth ed. Philadelphia: Lippincott Williams & Wilkins. 1305–1340.

[16] Schnell MJ, Mebatsion T, Conzelmann KK (1994) Infectious rabies viruses from cloned cDNA. EMBO J 13: 4195–4203. 7925265 10.1002/j.1460-2075.1994.tb06739.x PMC395346

[17] Bukreyev A, Lamirande EW, Buchholz UJ, Vogel LN, Elkins WR, et al (2004) Mucosal immunisation of African green monkeys (Cercopithecus aethiops) with an attenuated parainfluenza virus expressing the SARS coronavirus spike protein for the prevention of SARS. Lancet 363: 2122–2127. 15220033 10.1016/S0140-6736(04)16501-X PMC7112367

[18] Tompkins SM, Lin Y, Leser GP, Kramer KA, Haas DL, et al (2007) Recombinant parainfluenza virus 5 (PIV5) expressing the influenza A virus hemagglutinin provides immunity in mice to influenza A virus challenge. Virology 362: 139–150. 17254623 10.1016/j.virol.2006.12.005 PMC1995462

[19] Kawano M, Kaito M, Kozuka Y, Komada H, Noda N, et al (2001) Recovery of infectious human parainfluenza type 2 virus from cDNA clones and properties of the defective virus without V-specific cysteine-rich domain. Virology 284: 99–112. 11352671 10.1006/viro.2001.0864

[20] Kawano M, Okamoto K, Bando H, Kondo K, Tsurudome M, et al (1991) Characterizations of the human parainfluenza type 2 virus gene encoding the L protein and the intergenic sequences. Nucleic Acids Res 19: 2739–2746. 1645865 10.1093/nar/19.10.2739 PMC328195

[21] Niwa H, Yamamura K, Miyazaki J (1991) Efficient selection for high-expression transfectants with a novel eukaryotic vector. Gene 108: 193–199. 1660837 10.1016/0378-1119(91)90434-d

[22] Buchholz UJ, Finke S, Conzelmann KK (1999) Generation of bovine respiratory syncytial virus (BRSV) from cDNA: BRSV NS2 is not essential for virus replication in tissue culture, and the human RSV leader region acts as a functional BRSV genome promoter. J Virol 73: 251–259. 9847328 10.1128/jvi.73.1.251-259.1999 PMC103829

[23] Jung T, Lack G, Schauer U, Uberuck W, Renz H, et al (1995) Decreased frequency of interferon-gamma- and interleukin-2-producing cells in patients with atopic diseases measured at the single cell level. J Allergy Clin Immunol 96: 515–527. 7560664 10.1016/s0091-6749(95)70296-2

[24] Matsuyama T, Urano K, Ohkido M, Ozawa H, Ohta A, et al (1999) The quantitative and qualitative defect of CD4+ CD45RO+ memory-type T cells are involved in the abnormality of TH1 immunity in atopic dermatitis patients. Clin Exp Allergy 29: 687–694. 10231330 10.1046/j.1365-2222.1999.00568.x

[25] Reinhold U, Wehrmann W, Kukel S, Kreysel HW (1990) Evidence that defective interferon-gamma production in atopic dermatitis patients is due to intrinsic abnormalities. Clin Exp Immunol 79: 374–379. 2107991 10.1111/j.1365-2249.1990.tb08098.x PMC1534961

[26] Wu J, Xu J, Cai C, Gao X, Li L, et al (2009) Ag85B DNA vaccine suppresses airway inflammation in a murine model of asthma. Respir Res 10: 51. 19531238 10.1186/1465-9921-10-51 PMC2713210

[27] Zuany-Amorim C, Sawicka E, Manlius C, Le Moine A, Brunet LR, et al (2002) Suppression of airway eosinophilia by killed Mycobacterium vaccae-induced allergen-specific regulatory T-cells. Nat Med 8: 625–629. 12042815 10.1038/nm0602-625

[28] Kuwahara M, Yamashita M, Shinoda K, Tofukuji S, Onodera A, et al (2012) The transcription factor Sox4 is a downstream target of signaling by the cytokine TGF-beta and suppresses T(H)2 differentiation. Nat Immunol 13: 778–786. 22751141 10.1038/ni.2362 PMC3477402

